# A Dangerous Hospital

**Published:** 1890-06-21

**Authors:** 


The Hospital

A Weekly Journal of

Science, Medicine, Nursing, and Philanthropy.

*For Hospitals, Asylums, Medical Practitioners, Students, Nurses, Families, Parishes, Congregations, and the Charitable Public.*

Vol. VIII.—No. 195.] [June 21, 1890.

### A Dangerous Hospital.

A doctor should never be ill, and a hospital should never get out of order. All things considered, it is surprising how constantly well doctors manage to keep themselves, and how sanitarily sound hospitals generally are. But this rule of wholesome sanitation is not invariable; and when a hospital has become organically and radically defective, the questions arising therefrom are of the most serious import. The County Hospital at Derby has itself become a patient; and as a good many other hospitals may now and again be in like circumstances, we propose to hold with our readers a "consultation," here and now, in order that intelligent conclusions may be arrived at, and that sound precedents may be established for general use hereafter.

To begin with the "symptoms" first: A valuable and much-esteemed nurse sickened and died at the Derby Hospital. The medical officers, the nursing staff, and the managers were seriously and justly alarmed. Dr. Seaton was appointed to investigate the sanitary condition of the buildings. He reported such extensive disorganization of the sewage arrangements that it was felt to be imperative to have all the drainage thoroughly examined by competent and experienced architects. Messrs. Keith, Young, and Hall, who are well-known specialists in hospital construction, were accordingly called in. They discovered that the poisonous smells which had resulted in the illness and death of Head Nurse Cross were due to "culpable negligence in the construction of the sewers of the infirmary."

Here let us pause for a minute to consider what is the significance of culpable negligence in the construction of sewers belonging to infirmaries, private houses, or other places in which human beings have to live. The work of the drain-maker is out of sight; and if he wishes to be negligent he can be so without much risk of present detection. But what happens, sooner or later, as the result of his work? Nurse Cross dies at the Derby Hospital; at some other hospital, or private house, perhaps half-a-dozen nurses or children sicken or die. In many cases where no deaths occur unaccountable illnesses of various kinds break out, and the work of the institution often entirely fails of success where success would be easy if sanitation were good. Now, the responsibility for such insanitary dangers must be placed upon some one, and the person on whom to place it is the original constructor of the drains. His so-called "culpable negligence" is really shameful wickedness, and ought to be a criminal offence. It has resulted in the death of one or many innocent persons. True, the drain-maker was not an actual murderer, in that he did not intend to cause death. But the man who slays another by wilful negligence and carelessness is very little less guilty than a murderer, and should be punished accordingly. Penal servitude for a long term of years ought to be made imperative by statute in all such cases. We say these things deliberately because we have ourselves seen plumbers constructing drains in exactly the same way as the Derby drains seem to have been constructed. It is hardly necessary to add that those plumbers were promptly informed that they deserved to be hanged for their pains.

In dealing with the case of a hospital as distinguished from that of a patient, we have to bear in mind that the ills and defects of the former must be treated thoroughly, and cured completely—in other words, a hospital must be made entirely healthy and sanitarily whole. This is the most fundamental of all the conditions of the work and life of a hospital. Every manager should fix this elementary principle so firmly in his mind that it will govern and regulate all his other judgments and decisions that bear upon the questions of construction and management. Some of the members of the Derby Committee, as is quite natural, think that the present hospital will continue to serve its purpose if it is patched up and made reasonably wholesome in its sanitation; others are for pulling the building down root and branch, and for erecting a new one, constructed throughout on the most improved sanitary principles, either on the old site or elsewhere. That is the question which, of course, will always arise when serious sanitary or other structural defects are found in any particular hospital. The various members of a managing Board will look at the subject from their various points of view, and will give their decisions on very different grounds. Most of them, however, will be guided by the financial aspects of the case. But we point out, and insist that in hospital affairs, and especially those of a sanitary character, financial questions are, and must be, secondary and not primary. It is, above all things, imperative that a hospital have throughout a perfectly healthy atmosphere. Whatever the cost of securing this result, it must infallibly be secured. The question as to whether a given town is to have a healthy or an unhealthy hospital should never be so much as debated. The real point at issue should be—is there to be a healthy hospital, or is there to be no hospital at all?

This is the question which the Derby managers have now to face. The state of things disclosed by the inspection of Dr. Seaton, and the more searching investigations of Messrs. Young and Hall, have caused quite a consternation among both managers and officials. The in-patient department is practically at a standstill. Doctors and nurses have fled. A certain number of accidents and other casualties continue to demand treatment, and for these hospital tents either have been or are about to be erected. This promptitude is entirely to be commended: where health and life are at stake temporising measures are not to be thought of. And that health and life are really at stake at Derby there can be little question if Mr. Swingler, one of the members of the committee of investigation, be correctly reported. A sort of "rat fever" had, he said, been going over the institution, and he "could not help feeling that it ought to have been dealt with long ago. He had been told some time since by an observer who had been sitting at the bedside of a patient, that he could see the rats running about. Rats could not come to that extent in a hospital without their movements being in progress a long time, and the very fact of there being so many ought to have shown the staff that the communication was from the sewers. The statement from the architects was deplorable."

So far as the evidence available for outsiders goes, it seems to show the desirability of pulling down the present hospital at Derby and of building a new one. The radical defects of construction are so great that a sum of not less than £50,000 will, it is stated, be required for the proper reconstruction of the present building. On the other hand, for £60,000 an entirely new hospital of the most approved sanitary type, and with every modern improvement in structural details can be erected. Is it worth while, for the sake of £10,000 to renovate an old building, which may after all prove a failure, rather than to erect a new one for whose general excellence both the architects and the builders can be made responsible? The truth is that probably half the hospital buildings in the United Kingdom ought to be pulled down. The present generation of hospital managers are so thoroughly familiar with the principles of sanitary science that they forget how ignorant and careless their immediate predecessors were. As we have shown, the question of money is entirely secondary in importance. The really vital point is that of sanitation. Those managers who, from considerations of economy, decide to be content with what is less than the best, must remember that they cannot escape the responsibility of their own actions. The question at issue, it must be borne in mind, is not academical but practical; and the consequences that may follow upon mistaken judgment will also be practical. The members of the medical staff especially should realize their responsibility, and should stand immoveable on the ground of the highest sanitary excellence. The work of the hospital for the next two or three centuries may depend upon the decisions of the next two or three weeks. With such issues at stake that manager must hold himself both foolish and wicked who gives a judgment on any but the highest grounds.

